# Biochemical evaluation of molecular parts for flavonoid production using plant synthetic biology

**DOI:** 10.3389/fpls.2025.1528122

**Published:** 2025-04-15

**Authors:** Hyo Lee, Saet Buyl Lee, Sangkyu Park, Jaeeun Song, Beom-Gi Kim

**Affiliations:** Metabolic Engineering Division, National Institute of Agricultural Sciences, Rural Development Administration, Jeonju, Republic of Korea

**Keywords:** flavonoids, plant synthetic biology, molecular parts, enzyme activity, plant biofactory

## Abstract

Among organisms on Earth, plants have the unique ability to produce a wide variety of biomolecules using soil nutrients, air, and solar energy. Therefore, plants are regarded as the most productive and cost-efficient bioreactors among living organisms. Flavonoids, a major group of secondary metabolites exclusively produced in plants, play crucial roles in plant physiology and have various effects on human health. Flavonoids are used in diverse industries such as the pharmaceutical, nutraceutical, and cosmetics industries. These compounds are typically extracted from specific plants that naturally produce large amounts of the target flavonoid for commercial production. However, with the increasing demand for flavonoids, efforts have been made to enhance flavonoid production using synthetic biology for sustainable production in microbes or plants. Synthetic biology has been utilized for plant metabolic engineering to reconstitute the biosynthetic pathways of target flavonoids at the whole-pathway level, thereby enhancing flavonoid production. For the most efficient flavonoid production using plant synthetic biology, first of all, optimized molecular parts and enzymes must be identified and selected. The best modules to produce the precursors and final target flavonoids can then be constructed using these optimized parts. In this review, we summarize the enzyme kinetics of natural and engineered molecular parts derived from different plant species and provide insight into the selection of molecular parts, design of devices, and reconstitution of pathways based on enzyme performance for sustainable flavonoid production using plant synthetic biology.

## 1 Introduction

Plants, animals, and microbes consist of millions of chemicals, ranging from small molecules such as glucose and hormones to macromolecules such as proteins and nucleic acids. Plants serve as sustainable chemical factories because they utilize sunlight, CO_2_, nitrogen, and soil minerals to produce a diverse array of chemicals and supply them to organisms such as animals and microbes (Fesenko and Edwards, 2014; Birchfield and McIntosh, 2020).

Plants produce a wide variety of secondary metabolites that enable them to survive against herbivores and pathogens and harsh environmental conditions, as they are unable to move to avoid unfavorable environments and must endure these conditions. Consequently, plants produce a much wider range of chemicals than animals. Secondary metabolites not only function within plants but also have significant implications for human health. These compounds have garnered attention for use in pharmaceuticals and functional foods owing to their reported benefits. Plant secondary metabolites are classified into terpenes, flavonoids, N-containing compounds, benzenoids, phenylpropanoids, and others based on their chemical structures (Liga et al., 2023; Lin et al., 2023). The name “flavonoid” was derived from the Latin word “flavus”, meaning yellow. Typical flavonoid plant pigments contribute to the coloration of fruits and flowers. Moreover, flavonoids act as signaling compounds in plant–microorganism symbiosis and provide protection against abiotic stresses such as ultraviolet irradiation, as well as biotic stresses such as attack from herbivores, viruses, bacteria, and fungi (Kesarkar et al., 2009; Liga et al., 2023). Recent research has highlighted the various medical benefits of flavonoids, including anticancer, antioxidant, anti-inflammatory, and antiviral activities as well as activity against vascular disorders and antimutagenic properties. Consequently, flavonoids are extensively applied in the pharmaceutical, nutraceutical, and cosmetic fields due to their multifunctional bioactivities and therapeutic potential (Xu et al., 2023; Dini and Grumetto, 2022; Lee and Park, 2022).

Natural flavonoid production in plants might not meet market requirements owing to the limited range of suitable plant sources containing significant quantities of target flavonoids. Additionally, some flavonoid production methods, such as the synthesis of diosmin from hesperidin through oxidative reactions using catalysts and organic chemicals, may lead to environmental pollution. Thus, efforts have been made to produce flavonoids in microbes such as the bacterium *Escherichia coli* or the yeast *Saccharomyces cerevisiae* for sustainable production (Okoye et al., 2023; Pandey et al., 2016). Microbes have been successfully used as platforms to reconstitute metabolic pathways to produce valuable flavonoids, although this approach may not be economically viable compared to flavonoid production from plant materials. Challenges include the requirement for subcellular compartments and specific tissues for the functioning of plant enzymes in microbes. Moreover, microbial production requires large-scale fermenters and nutrient sources for microbial growth (Kotopka et al., 2018; Isogai et al., 2022; Birchfield and McIntosh, 2020).

By contrast, plants might represent an ideal platform for the large-scale production of nutraceuticals and pharmaceuticals through synthetic biology, despite existing technological bottlenecks. Recent advances in plant synthetic biology are enabling the development of crops as practical platforms for the targeted production of valuable chemicals derived from medicinal plants in large quantities. For example, (-)-deoxypodophyllotoxin, a precursor of the anticancer drug podophyllotoxin, which was originally derived from Himalayan mayapple, was successfully produced at a scale of milligrams per gram of dry weight in *Nicotiana benthamiana* leaves via transient expression of 16 genes encoding enzymes in the etoposide aglycon biosynthetic pathway (Schultz et al., 2019). Other pharmaceuticals including taxdiene, disgenin, and diosmin have also been successfully produced in *N. benthamiana* using synthetic biology (Zhu et al., 2021; Lee et al., 2024). However, plants developed for the production of flavonoids or high-value metabolites using synthetic biology cannot be cultivated in the field in several countries owing to GMO regulations. Alternatively, the integration of in-door farm, plant cell culture and hairy root culture system and synthetic biology, referred to as plant biofactory allows medically or industrially important chemicals including diverse materials to be produced in crops under completely controlled environmental conditions. In the USA and some other nations, GM plants developed using synthetic biology might be cultivated in fields if the products and ingredients are healthy and safe for human and environment. Those approaches blur the boundaries between the agricultural and pharmaceutical industries and accelerates advancements in plant synthetic biology.

The genes, promoters, and terminators used in plant synthetic biology are referred to as parts, and those consist of functionally minimal units, which are organized into modules (Kotopka et al., 2018; Lin et al., 2023; Vazquez-Vilar et al., 2023). Plant synthetic biology, combined with metabolic engineering, aims to produce natural products through biochemical reactions involving several substrates, intermediates, target products, and enzymes. The production yield of plant natural products is influenced by the effective selection and assembly of genetic parts, commonly known as DNA sequence. The performance of many parts and metabolite synthesis modules is determined by enzyme activities and substrate specificities. Thus, in this review, we describe the performances of molecular parts related to five flavonoid backbone biosynthesis (flavanone, flavone, flavonol, dihydroflavonol and anthocyanin) in terms of their enzymatic characteristics, such as enzyme activity and substrate specificity. We collected data and adjusted the units from as many *in vitro* assays of enzymes involved in the five flavonoid backbone biosynthesis as possible in **Supplementary Tables S1**; **S2** and listed enzymes with the highest or special characteristics in **Tables 1**, **2**. We hope this information will help researchers choose the most efficient molecular parts for flavonoid production.

**Table 1:** Parts for the upstream pathway of flavonoid with high efficiency enzymatic activity.

Enzymes	Gene name	Species	ID	Substrates	K_m_ (μM)	V_max_ (nKat·mg^-1^)	Kcat	K_cat_/K_m_ (M^-1^·S^-1^)	V_max_/K_m_	References
PAL	AtPAL2	*Arabidopsis thaliana*	At3g53260	L-Phe	64	10.5	3.20	50,000		Cochrane et al., 2004
	PAL4	*Nicotiana tabacum*	EU883669/70	L-Phe	52.4	19.6	1.53	29,198		Reichert et al., 2009
	PyPAL1	*Pinus yunnanensis*	OR714894	L-Phe	1.861 (mmol·L^-1^)	28.8			15.48	Mu et al., 2024
	SbPAL1	*Sorghum* *bicolor*	Sb04g026510	L-Phe	340		1.76	5,176		Jun et al., 2018
	ZmPAL1	*Zea may*	L77912	L-Phe	658		11.90	18,085		Rösler et al., 1997
C4H	GmC4H14	*Glycine max*	Glyma.14G205200	trans-Cinnamic acid	2.74	0.94			0.34	Khatri et al., 2023
	SbC4H1	*Sorghum* *bicolor*	Sobic.002G126600	trans-Cinnamic acid	0.61	12.1 (min^-1^)			19.84	Zhang et al., 2020
	PbC4H1	*Pyrus bretschneideri*	Pbr013141.1	trans-Cinnamic acid	10.23					Li et al., 2020b
	LaeC4H	*Leucojum aestivum*	UIP35210	trans-Cinnamic acid	1.21	0.12 (μM·min^-1^)			0.10	Karimzadegan et al., 2024
	PtrC4H1 +PtrC4H2	*Populus trichocarpa*	POPTR_0013s15380+POPTR_0019s15110	trans-Cinnamic acid	3.69	1652.83			447.92	Chen et al., 2011
4CL	At4CL1	*Aarabidopsis thaliana*	At1g51680	p-Coumaric acid	6	61.3	3.96	660,000		Costa et al., 2005
	Os4CL3	*Oryza sativa L. ssp* japonica	Os02g08100	p-Coumaric acid	4.9	4.7	0.29	58,163		Gui et al., 2011
	Gm4CL3	*Glycine max*	AF002258	p-Coumaric acid	9	100 (relative % of coumarate)			11.12	Lindermayr et al., 2002

**Table 2:** Parts charcterized biochemically for flavonoid biosynthetic pathway with high enzyme activities.

Enzymes	Gene name	Species	ID	Substrates	K_m_ (μM)	V_max_ (nKat·mg^-1^)	K_cat_ (S^-1^)	K_cat_/K_m_ (M^-1^·S^-1^)	V_max_/K_m_	References
CHS	GbCHS	*Ginko biloba*	AY647263	p-coumaroyl-CoA	4.29		0.113	26,379		Waki et al., 2020
	SmCHS	*Selaginella moellendorffii*	270496	p-coumaroyl-CoA	3.15		0.06567	20,847		
CHI	GmCHI2	*Glycine max*	Glyma.20G241700	Naringenin chalcone	2.00		478.800	239,400,000		Ralston et al., 2005
	DaCHI1	*Deschampsia antarcita*	FR714890.1	Naringenin chalcone	8.50		130.31667	15,331,373		Park et al., 2018
F3H	OsF3H1	*Oryza sativa*	NM_001060692 Os02g0767300	Eriodictyol	57.80		0.210	3,633		Kim et al., 2008
	AtF3H /TT6	*Arabidopsis thaliana*	At3g51240	Naringenin	24.00	0.000002			0.00000007	Owens et al., 2008
FNSI	CjFNSI1	*Conocephalum japonicum*	MK557768	Naringenin	9.40		0.250	26,596		Li et al., 2020a
				DHK	13.00		0.300	23,076		
	CjFNSI1 /F2H	*Conocephalum japonicum*	MK557767	Naringenin	14.00		0.170	12,143		
				DHK	38.00		0.042	1,105		
	PcFNSI	*Petroselinum crispum*	AY817680	Naringenin	0.31		0.027	87,097		
FNSII	LjFNSII /F2H-1.1	*Lonicera japonica*	KU127576	Naringenin	9.93	13.830			1.393	Wu et al., 2016
				Eriodictyol	5.07	22.270			4.393	
				Liquiritigenin	6.48	38.390			5.924	
	LjFNSII /F2H -2.1	*Lonicera japonica*	KU12`7578	Naringenin	1.63	2.420			1.485	
				Eriodictyol	2.05	8.200			4.000	
				Liquiritigenin	2.56	10.310			4.027	
	LmFNSII /F2H -1.1	*Lonicera macranthoides*	KU127580	Naringenin	1.63	1.730			1.061	
				Eriodictyol	3.09	3.830			1.239	
				Liquiritigenin	2.38	6.140			2.580	
FLS	AcFLS -HRB	*Alium cepa*	GeneBank KY369210	DHK	15.52		0.002	155		Park et al., 2017
				DHQ	26.32		0.021	802		
	ZmFLS1	*Zea mays*	BT039956	DHK	58.40		6.600	113,014		Ferreyra et al., 2015
				DHQ	151.10		3.900	25,811		
	RcFLS1	*Rubus chingii*	LG02.1317	DHK	33.90		0.075	2,209		Lei et al., 2023
				DHQ	56.90		0.149	2,617		
				Naringenin	43.60		0.005	103		
				Eriodictyol	34.50		0.014	414		
DFR	CsDFRa	*Camelina sinensis*	KY615690	DHK	145.10		5.410	37,285		Ruan et al., 2022
				DHQ	41.80		10.390	248,565		
				DHM	58.44		10.470	179,158		
	DFR1 ES	*Fragaria Xananassa* cv. *Elsanta*	KC894048	DHK	0.40	11.400			28.500	Miosic et al., 2014
	DFR2 ES	*Fragaria Xananassa* cv. *Elsanta*	KC894055	DHQ	0.40	3.100			7.750	
				DHM	2.30	11.200			4.870	

## 2 Molecular parts of the phenylpropanoid biosynthesis pathway for flavonoid production

### 2.1 Phenylalanine ammonia lyase/tyrosine ammonia lyase

**Figure 1:**
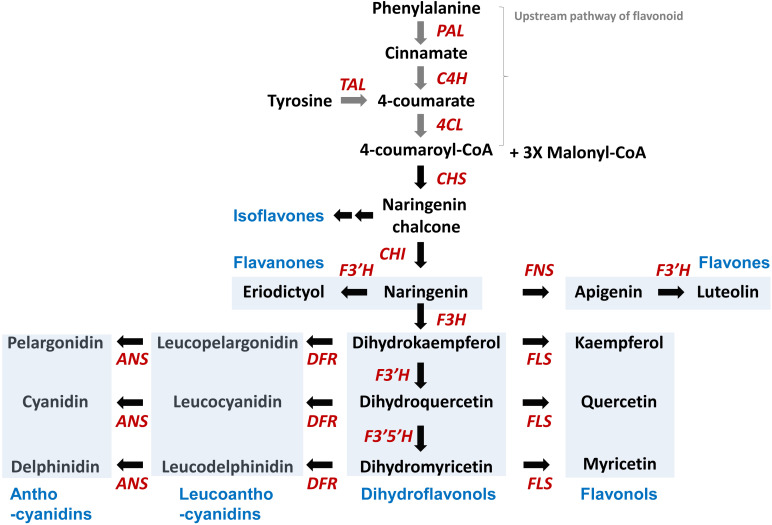
Biosynthetic pathway for the five flavonoid subclasses starting from phenylalanine. PAL, phenylalanine ammonia lyase; C4H, cinnamate 4-hydroxylase; 4CL, 4-coumarate CoA ligase; CHS, chalcone synthase; CHI, chalcone isomerase; F3′H, flavonoid 3′-hydroxylase; F3H, flavanone 3-hydroxylase; FNS, flavone synthase; FLS, flavonol synthase; DFR, dihydroflavonol 4-reductase; ANS, anthocyanidin synthase. Blue boxes highlight examples of flavonoid subclasses, such as isoflavones, flavanones, flavones, flavnonols, dihydroflavnonols, lecuanthocyanidins, and anthocyanidins.

Flavonoid synthesis begins from phenylalanine or tyrosine via the phenylpropanoid pathway (**Figure 1**). Phenylalanine ammonia lyase (PAL) and tyrosine ammonia lyase (TAL) catalyze the removal of the ammonia groups from amino acids by cleaving carbon–nitrogen bonds. Phenylalanine and tyrosine are converted to *trans*-cinnamic acid and 4-coumarate through deamination by PAL activity and TAL activity, respectively. These enzymes form tetramers and are inhibited by their product, *trans*-cinnamic acid. Plants generally have monofunctional PAL activity, which shows approximately 1,000-fold higher enzyme efficiency for phenylalanine over tyrosine (Cochrane et al., 2004). However, a monofunctional TAL has not yet been identified in plant. Instead, bifunctional phenylalanine/tyrosine ammonia lyases (PTALs) have been reported in monocot grass family Poacease such as maize (*Zea mays*), Brachypodium, and rice (*Oryza sativa*) (Jun et al., 2018; Barros and Dixon, 2020). In this review, we aim to summarize only PAL activities derived from plants. *In vitro* PAL activities measured in several plants range from 39 to 50,000 as measured by K_cat_/K_m_ (S^−1^·M^−1^) (**Supplementary Table S1**) (Cochrane et al., 2004; Li et al., 2023). Among these, Arabidopsis (*Arabidopsis thaliana*) AtPAL2 has the highest enzyme efficiency, although it is difficult to compare enzyme activities exactly among different species because it depends on reaction conditions (**Table 1**).

PAL activity measured in the same species under the same enzyme assay conditions vary greatly. For example, four different PAL activity values in Arabidopsis have been reported, with an approximately 2-fold difference in enzyme efficiencies, even when those with very low efficiency were excluded. On the other hand, enzyme engineering targeting residues suggested to play key roles in substrate selectivity between PAL and TAL successfully altered the enzyme activity and substrate specificity of PAL (Barros and Dixon, 2020). Mutating Phe144 to His in Arabidopsis PAL increased TAL activity up to 18-fold and decreased PAL activity up to 80-fold (Watts et al., 2006). The H123F mutation of *Sorghum bicolor* PAL1 (SbPAL1) enhanced the catalytic efficiency for Phe up to 6.2-fold and disrupted the catalytic activity for Tyr (Jun et al., 2018). Additionally, the mutation of ZmPAL2 improved the catalytic activity up to 4.5-fold (Zheng et al., 2024). Thus, optimized PAL enzymes can be selected for each reconstituted pathway and applied as parts for plant synthetic biology.

### 2.2 Cinnamate 4-hydroxylase

*Trans*-cinnamate, a compound biosynthesized by PAL, is converted into *p*-coumaric acid/4-hydroxy cinnamic acid in plants by the cytochrome P450 cinnamate 4-hydroxylase (C4H), a member of the CYP73A family. The C4H gene family typically has a low copy number, usually ranging from one to five members in the plant genome (Kumar et al., 2013). C4Hs localize to subcellular membranes in the endoplasmic reticulum (ER), serving as a nucleation point to form a multi-enzyme complex with PAL and 4-coumarate-CoA ligase (4CL) known as the phenylpropanoid metabolon. The electrons necessary for catalysis by C4H are provided by NADPH cytochrome P450 reductase (CPR), which is colocalized with C4H on the exterior surface of the ER membrane (Zhang et al., 2020). The K_m_ values of C4H derived from several plant species for *trans*-cinnamic acid *in vitro* range from 0.61 to 40.68 µM (**Supplementary Table S1**). The formation of a complex with 4CL improved the enzyme activity more than 100-fold in *Populus trichocarpa* (**Table 1**) (Chen et al., 2011). The formation of a C4H and 4CL complex might be critical for improving the production of phenylpropanoid pathway products. Indeed, expressing all genes in the phenylpropanoid pathway improved the production of downstream metabolites (Schultz et al., 2019; Lee et al., 2024).

### 2.3 4-coumarate-CoA ligase

4CL catalyzes the formation of *p*-coumaroyl-CoA by attaching coenzyme A (CoA) to *p*-coumaric acid. The 4CL enzymes exist in several isoforms and are encoded by multiple genes within a species (Lavhale et al., 2018). These enzymes have been identified and functionally characterized in various plant species. Plant 4CLs are classified into four groups: I, II, III, and IV. Dicot plants contain group I and II 4CLs, while monocot plants contain group III and IV 4CLs. Group II and IV 4CLs are mainly involved in flavonoid biosynthesis, whereas group I and III 4CLs are involved in lignin biosynthesis. 4CLs have a broad substrate spectrum and function at a crucial branchpoint that determines the biosynthesis of lignin, flavonoids, or other phenylpropanoid derivatives. Arabidopsis 4CLs use diverse substrates such as cinnamic acid, *p*-coumaric acid, caffeic acid, and ferulic acid, and the enzyme kinetics for diverse substrates vary. Among the five Arabidopsis 4CLs, At4CL3 shows much higher enzyme efficiencies for 4-coumaric acid than for other substrates (Costa et al., 2005). In rice, Os4CL3 has the highest enzyme efficiency for 4-coumaric acid (**Supplementary Table S1**) (Gui et al., 2011). Thus, Os4CL3 and At4CL3 might be good candidate molecular parts for flavonoid production (**Table 1**). These three enzymes (PAL, C4H, and 4CL) are key components of the phenylpropanoid biosynthesis pathway to generate flavonoids. The formation of complexes by these enzymes can lead to different enzyme performances and can be engineered to increase biomass production via the regulation of lignin production. Therefore, their co-expression might improve the production of phenylpropanoid pathway products (Schultz et al., 2019; Lee et al., 2024).

## 3 Molecular parts for the production of basic flavonoid backbones

**Figure 2:**
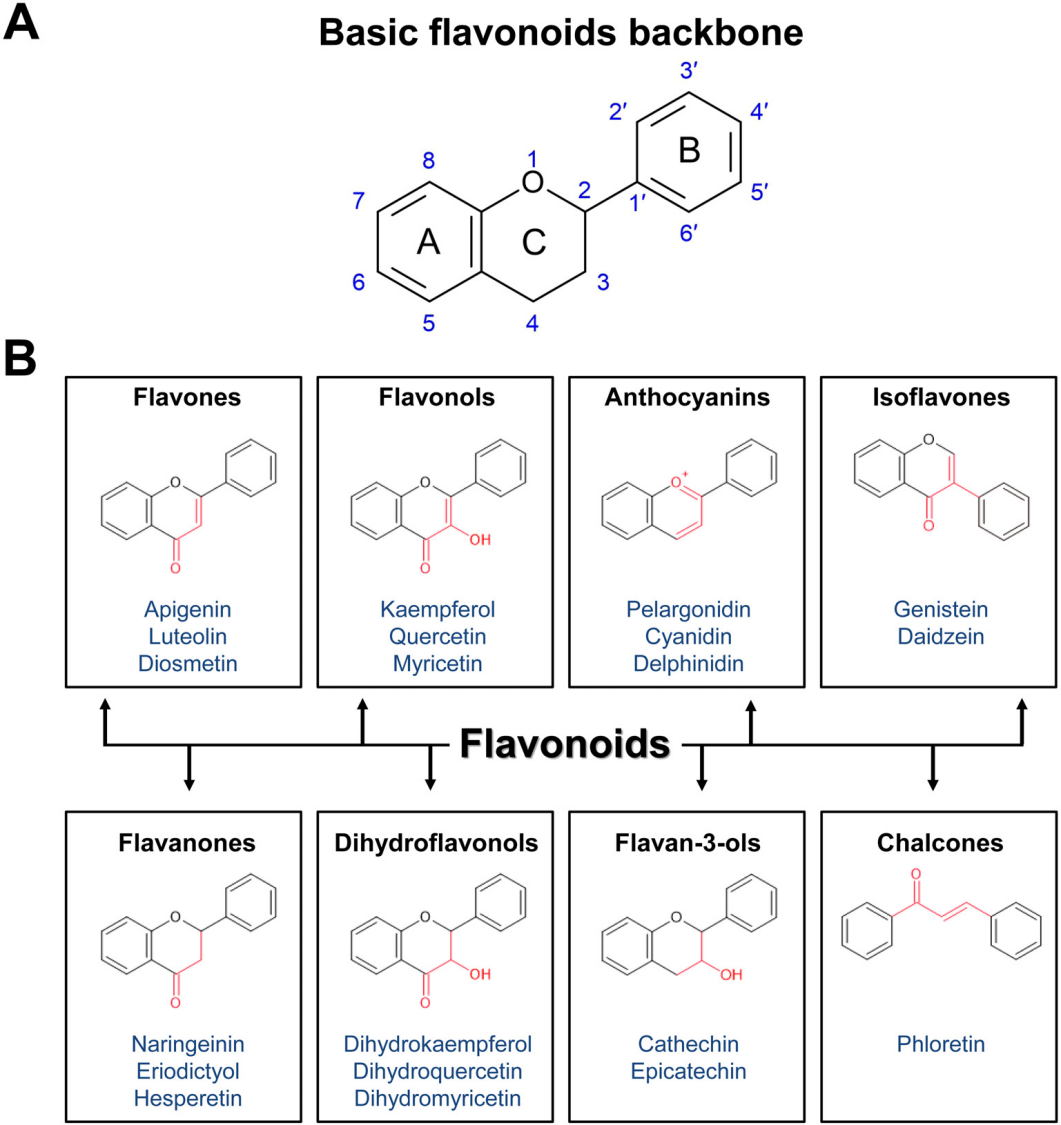
Basic structure and classification of flavonoids. **(A)** Basic structure of flavonoids. **(B)** Classification of flavonoids based on chemical structures, with examples listed. Flavonoids are classified into eight subclasses: flavones, flavonols, anthocyanins, isoflavones, flavanones, dihydroflavonols (flavanonols), flavan-3-ols (flavanols), and chalcones.

The basic chemical structure of flavonoids consists of a 15-carbon (C6-C3-C6) skeleton and two benzene rings, A and B, connected by a three-carbon bridge, which usually form the heterocyclic ring C. To date, more than 8,000 flavonoid compounds have been identified in nature (Mutha et al., 2021). Flavonoids are classified into different subgroups based on the saturation and oxidation states of ring C and the hydroxylation of rings B and C. These subgroups include flavones, flavonols, flavanones, isoflavones, anthocyanidins, flavan-3-ols, and chalcones (**Figure 2**). Here we describe the kinetics of biosynthetic enzymes involved in flavonoid backbone production for chalcones, flavones, flavonols, anthocyanin and flavanones, including the following: chalcone synthase (CHS), chalcone isomerase (CHI), flavone synthase (FNS), flavanone 3-hydroxylase (F3H), flavonol synthase (FLS), and dihydroflavonol 4-reductase (DFR).

### 3.1 Chalcone synthase

CHS, a class of Type III polyketide synthases (PKSs), catalyzes the first step of the flavonoid biosynthesis pathway. One molecule of *p*-coumaroyl-CoA and three molecules of malonyl-CoA are condensed by CHS to synthesize one molecule of naringenin chalcone (4,2′,4′,6′-tetrahydroxychalcone). The first plant *CHS* gene to be isolated and identified was from parsley (*Petroselinum crispum*). Subsequently, *CHS* genes have been functionally identified in more than 20 plant species (Abe and Morita, 2010; Dao et al., 2011). The plant-type III PKSs share 30–95% amino acid sequence identity. CHS and its homologs catalyze the conversion of a variety of CoA-linked starter substrates, ranging from aliphatic-CoA to aromatic-CoA and from small acetyl-CoA and polar malonyl-CoA to nonpolar *n*-hexanoyl-CoA substrates. For example, Jez et al. (2001) reported that CHS2 from alfalfa (*Medicago sativa*) has quite high and similar enzyme efficiencies for *p*-coumaroyl-CoA and several substrates with CoA functional groups, such as malonyl-CoA and feruloyl-CoA. CHS is a promiscuous enzyme that catalyzes the formation of other polyketides, at least *in vitro*. This catalytic promiscuity provides an important basis for the adaptive evolution of plant specialized metabolism. CHS can be used as a key molecular part in the reconstitution of metabolic pathways to synthesize diverse and novel chemicals, depending on the substrates. Waki et al. (2020) compared the activities of CHS isolated from five species: PpCHS from *Physcomitrella patens*, SmCHS from *Selaginella moellendorffii*, GbCHS from *Gingko biloba*, OsCHS-1 from *Oryza sativa*, and AmCHS from *Antirrhinum majus*. The efficiencies of these enzymes for *p*-coumaroyl-CoA differed by up to 17.8-fold (**Supplementary Table S2**). Among these, GbCHS and SmCHS exhibited high catalytic efficiencies for naringenin chalcone production from *p*-coumaroyl-CoA (**Table 2**). Thus, selecting a CHS with high enzyme efficiencies is crucial for improving naringenin chalcone production, and GbCHS and SmCHS could be used as molecular parts for synthetic biology.

### 3.2 Chalcone isomerase

CHI catalyzes the stereospecific cyclization of chalcones to (2*S*)-flavanones, such as naringenin (2*S*-5,7,4′-trihydroxyflavanone) (Lewis et al., 2024). CHI performs this reaction much more efficiently than the spontaneous cyclization reaction, which produces both 2*S*- and 2*R*-isomers. Only 2*S*-isomers, which act as biological precursors for flavonoid biosynthesis in nature, are produced through catalysis by CHI (Bednar and Hadcock, 1988). The CHI superfamily comprises four types: I, II, III, and IV (Park et al., 2018; Ralston et al., 2005). Type I CHI enzymes, which are ubiquitous in vascular plants, exhibit typical CHI catalytic activity, converting naringenin chalcone to (*2S*)-naringenin. Type II CHI enzymes (which are specific to legumes) have broader substrate specificity, catalyzing the conversion of isoliquiritigenin (6′-deoxychalcone) to 2*S*-liquiritigenin (5-deoxyflavanone) as well as naringenin chalcone (6′-hydroxychalcone) to 2*S*-naringenin (5-hydroxyflavanone) (Cheng et al., 2018). Type III CHI enzymes lack chalcone cyclization activity but possess fatty acid binding properties, influencing fatty acid biosynthesis and storage in developing Arabidopsis embryos (Ngaki et al., 2014). Type IV CHI enzymes lack catalytic activity but can enhance flavonoid production and flower pigmentation (Morita et al., 2014). CHI-like protein (CHIL) binds to CHS and enhances CHS-catalyzed THC production, resulting in improved metabolic flux from the general phenylpropanoid pathway to the flavonoid pathway (Waki et al., 2020).

Cheng et al. (2018) compared the activities of CHIs isolated from five different plants. The efficiencies of these enzymes for naringenin chalcone ranged from 49.8 × 10^6^ to 122 × 10^6^, representing a 2.4-fold difference (**Supplementary Table S2**). Among these enzymes, MpCHI isolated from *Marchantia paleacea* exhibited the highest enzyme efficiency. Ralston et al. (2005) reported the enzyme kinetics of three soybean (*Glycine max*) CHIs (CHI1A, CHI1B2, and CHI2) for seven chalcone substrates with different degrees of hydroxylation. GmCHI2, a type I CHI, uses only naringenin chalcone (4,2′,4′,6′-tetrahydroxychalcone) as a substrate. Two CHI type II enzymes, GmCHI1A and GmCHI1B2, have similar enzyme efficiencies for seven different chalcone substrates. In addition, *Deschampsia antarctica* CHI (DaCHI) exhibits strong substrate preference for naringenin chalcone (type I substrate) but weak substrate preference for isoliquiritigenin (type II substrate) (Park et al., 2018). Of the four OsCHIs examined in rice (OsCHI1, OsCHI3, OsCHI6, and OsCHI7), only type I OsCHI3 exhibited CHI activity for naringenin chalcone. However, OsCHI1 activity for isoliquiritigenin was not detected (Park et al., 2021). Thus, CHI enzymes, which show promiscuity for diverse substrates, play important roles in increasing the diversity of flavonoids together with CHS. Among CHI enzymes mentioned above, GmCHI2 showed the highest enzyme efficiency for naringenin chalcone, followed by DaCHI1. Thus, these two enzymes could be selected to improve naringenin production (**Table 2**).

### 3.3 Flavone synthase

The evolution of land plants was accompanied by the emergence of different classes of flavonoids. Liverworts, the most primitive land plants, utilize FNSI/flavanone 2-hydroxylase (F2H) to produce flavones and 2-hydroxyflavones. FNSI/F2H evolved into FNSI/F3H in moss. Such a transition further developed in gymnosperms, and the enzyme completely shifted to the bona fide F3H in angiosperms. FNSI has been lost in most angiosperms, as its role in flavone production was replaced by the cytochrome P450 monooxygenase FNSII enzymes that have emerged in angiosperms (Li et al., 2020a; Du et al., 2016). However, distinct FNSIs have emerged through duplication and substitution events of F3H in Apiaceae plants (Gebhardt et al., 2005, 2007). Besides Apiaceae FNSIs, additional FNSIs have arisen independently from different lineages in *O. sativa*, *Z. mays*, and Arabidopsis. FNS converts flavanones to flavones by forming a double bond between C2 and C3 of flavanones. FNSI, a member of the 2-oxoglutarate-dependent dioxygenase (2-ODD) superfamily, requires a non-heme ferrous iron, 2-oxoglutarate, and O_2_ for the catalytic reaction, while FNSII is a member of the CYP93 subfamily (Martens and Mithöfer, 2005). Most FNSIIs in monocots and dicots belong to the CYP93G and CYP93B subfamilies, respectively (Akashi et al., 1999). Among FNSIs, although the primitive liverwort (*Conocephalum japonicum*) CjFNSI/F2Hs show promiscuous characteristics, exhibiting FNS, F2H, and FLS activities and relatively high substrate-binding affinity, FNSI (PcFNSI) from the Apiaceae family member parsley shows the highest catalytic efficiency owing to its overwhelmingly high binding affinity for naringenin. Among FNSIIs, *Lonicera* (honeysuckle) FNSIIs show the highest V_max_ values, and they prefer eriodictyol to naringenin as a substrate for their FNS activity (**Table 2**), suggesting that they are suitable for 3′,4′-dihydroxyflavone production.

### 3.4 Flavanone 3-hydroxylase

F3H converts flavanones such as naringenin, eriodictyol, and pentahydroxyflavanone to the dihydroflavonols dihydrokaempferol (DHK), dihydroquercetin (DHQ), and dihydromyricetin (DHM), respectively. Dihydroflavonols serve as common precursors for three major classes of end products: flavonols, anthocyanins, and proanthocyanidins (Park et al., 2019; Lei et al., 2023). Thus, F3H plays a crucial role as a key branchpoint in the flavonoid biosynthesis pathway. F3H belongs to the 2-ODD superfamily, which also includes FLS, anthocyanidin synthase (ANS), FNSI, and flavonol 6-hydroxylase (Wang et al., 2021). The 2-ODDs catalyze a variety of oxidation reactions (such as hydroxylation, desaturation, and oxidative ring closure) in plants, animals, and microorganisms, participating in a diverse array of primary and specialized metabolic pathways (Cheng et al., 2014). Among the three rice F3Hs, OsF3H1 has the highest enzyme efficiency for eriodictyol, as it is 1,000 times more efficient than OsF3H2 and ~100 times more efficient than OsF3H3 (Kim et al., 2008). Although enzyme activities were not characterized, several F3H gene and promoter combinations were tested to produce DHQ in yeast, with *Citrus sinensis* F3H (CsF3H) showing the highest activity (Yu et al., 2022). Studies of F3H activity from three different species (AtF3H, *Arabidopsis thaliana*; CtF3H, *Carthamus tinctorius*; GmF3H, *Glycine max*) for naringenin or eriodictyol substrate (Owens et al., 2008; Tu et al., 2016; Kim et al., 2008) revealed that AtF3H had the lowest K_m_ value of the three (**Supplementary Table S2**). Although enzyme activity data are lacking, OsF3H1 can be used to convert eriodictyol to DHQ (**Table 2**).

### 3.5 Flavonol synthase

Along with FNSI, F3H, and ANS, FLS belongs to the 2-ODD superfamily. Phylogenetic analysis suggested that the divergence of F3H, ANS, and FLS preceded the split of gymnosperms and angiosperms and that FLS emerged most recently (Choudhary and Pucker, 2024). FLS was first identified in parsley suspension cell cultures and was subsequently characterized in various species, such as tea plant (*Camellia sinensis*), *Zea mays*, and *Gingko biloba* (**Table 2**). FLS catalyzes the oxidation of the C-ring of dihydroflavonols (DHK, DHQ, and DHM) to generate flavonols (kaempferol, quercetin, and myricetin, respectively). Several FLSs are bifunctional, exhibiting both F3H and FLS activities (Chua et al., 2008; Park et al., 2023). Based on studies of Arabidopsis FLS1, five residues were shown to be involved in the binding of dihydroflavonol substrates. Among them, H132, which interacts with the B-ring hydroxyl group of the substrate, is thought to determine substrate preference. H132 allows FLS to accept all three types of dihydroflavonol substrates. This residue has been substituted with Y in gymnosperms and some monocots, and F in most other monocots, which allows FLSs to preferentially accept DHK and DHQ, respectively (Choudhary and Pucker, 2024). According to the available enzyme kinetics data, *Zea mays* FLS (ZmFLS) with an F132 residue shows strong catalytic efficiency among FLSs, with a superior V_max_ value for dihydroflavonol substrates. Its most preferred substrate is DHK, whereas FLS from the monocot onion (*Allium cepa*; AcFLS-HRB), which harbors Y132, exhibits a preference for DHQ over DHK (**Supplementary Table S2**). Therefore, the position corresponding to H132 might be an important factor in determining the flavonol product. The kinetics data show the F3H activities of *Ornithogalum caudatum* FLS1 (OcFLS1) and *Rubus chingii* FLS1 (RcFLS1), which are available to convert flavanones to dihydroflavonols (**Table 2**), indicating that the underlying genes could be employed for metabolic engineering to improve the efficiency of flavonol production.

### 3.6 Dihydroflavonol 4-reductase

DFR catalyzes reduction of dihydroflavonols (DHK, DHQ, and DHM) to form leucoanthocyanidins (leucopelargonidin, leucocyanidin, and leucodelphinidin, respectively), which are then converted to colored anthocyanidins by leucoanthocyanidin dioxygenase/anthocyanin synthase (LDOX/ANS). Proanthocyanidins first appeared in lycophytes, a group of seedless vascular plants. However, phylogenetic analysis showed that the ancient ancestor of DFR can be traced back to moss (Campanella et al., 2014). DFRs, which belong to the short-chain dehydrogenase family, require NAD(H) or NADP(H) as a cofactor. To date, most DFRs have been isolated from flowering plants, and their characteristics have been elucidated. DFR sequences harbor a conserved NADPH-binding domain and a substrate-binding domain. The third residue within the 26-amino-acid substrate-binding domain determines the substrate specificity of DFR. Monocots commonly contain an N residue at that position, which allows DFR to accept all three types of dihydroflavonols as substrates, whereas most dicots have D or A as well as an N residue. The D residue confers preferences for DHQ and DHM, while the A residue confers a strong preference for DHK. *Camellia sinensis* DFRa (CsDFRa), with a substrate specificity–determining N residue, strongly prefers DHQ and DHM over DHK. Strawberry (*Fragaria* × *ananassa*) ‘Elsanta’ DFR1 and DFR2 (DFR1 ES and DFR2 ES), which harbor an A and N residue, respectively, and show strikingly low K_m_ values for DHK and DHQ, respectively, compared to other DFRs (**Table 2**). To maximize the efficiency of producing anthocyanin-derived end products in plants using DFR, an elaborate strategy is required to avoid overlapping between the substrate preferences of FLS in the host plant and the DFR utilized, which could mitigate the competition between these enzymes for dihydroflavonol substrates.

## 4 Perspectives on strategies for developing molecular parts for flavonoid production in plant biofactory

To efficiently produce flavonoids in plants using plant synthetic biology, it is crucial to develop molecular parts with high enzyme activity and substrate specificity (Tohge et al., 2017). We propose two strategies for developing molecular parts for flavonoid production in terms of enzyme activity. One strategy is to select the best enzyme from diverse species. This involves cloning target enzymes from various plant sources, comparing their biochemical properties, and selecting the best-performing enzyme. The use of reported data for enzyme kinetics or a database for enzyme activity would help simplify this process. The second strategy is to engineer superior enzymes with the best activity and substrate specificity. To develop enzyme with the best activity that can be commercially utilized, recent approaches involve the large mutant library generation and automated high-throughput screening system such as biofoundry (Paddon et al., 2013). Optimized enzymes are applied as parts consisting of new metabolic pathway to produce flavonoid maximally in plants (Zha et al., 2019). Since the protein structures of flavonoid biosynthesis–related enzymes have already been identified, prediction and modeling could be used to optimize these enzymes. Recently the machine learning algorism named as UniKP was developed to predict the Km and Kcat values of enzymes based on amino acid sequences. And this algorism was used for directed evolution of TAL enzymes (Yu et al., 2023).

The secondary metabolites produced through the phenylpropanoid metabolic pathway can occasionally confer toxicity to plants or have a negative impact on their growth. For examples, excessive accumulation of anthocyanins has been reported to inhibit plant growth. As a major branch of phenylpropanoid metabolism, anthocyanin biosynthesis is closely linked to the lignin pathway (Shi and Xie, 2014). It has been reported that the activation of anthocyanin biosynthesis leads to changes in metabolic flux, negatively affecting the lignin pathway and causing growth deficiency in plants (Li et al., 2018). In addition, anthocyanin accumulation protects plants from high light stress but reduces photosynthesis. This leads to lower carbon assimilation, altered carbon-nitrogen metabolism, and decreased levels of key photosynthetic metabolites, which can ultimately inhibit plant growth (Zhao et al., 2022). To enhance the content of target compounds while minimizing the impact on plant growth and development, elaborate engineering of feedback inhibition is required. This includes regulating gene expression at specific times or in specific tissues using inducible or tissue-specific promoters, manipulating metabolic flux to suppress side pathways or competing metabolic routes, compartmentalizing enzymes, and utilizing transporter proteins to direct the accumulation of target compounds in specific organelles such as vacuoles or the apoplasts (Lin et al., 2023).

With revolutionary advances in metabolic engineering and synthetic biology technologies, the use of heterologous plants to produce natural biomolecules has become a promising alternative (Scown and Keasling, 2022).

Microbial systems, currently the predominant platform, offer several advantages, including rapid production, ease of genetic manipulation, and relatively straightforward processes for the separation and purification of the produced substances. However, these systems often entail higher initial investment costs and unit production costs compared to plant-based systems (Carvens et al., 2019; Pyne et al., 2019; Tusé et al., 2014; Wu et al., 2021). In large-scale production, plants do not require any expensive facilities such as fermenters, and plants can produce substrates required to biosynthesis secondary metabolites using light, water, and minerals, environmental friendly. However, slow growth can negative impact on large scale production because it requires time to fit specific growth stage for overexpressing genes through transient expression system or getting enough amounts of target compounds from transgenic plants. However, the presence of complex endogenous compounds in plants can present a significant bottleneck during the purification process. In large scale production, various steps are required to get target metabolites without byproducts or the endogenous compounds. Relatively low extraction efficiency was reported, only 0.1-1% of the pure target compounds was harvested using the plant system such as plant cell culture (Appelhagen et al., 2018) and transient expression system (Reed et al., 2017). Despite these challenges, plant systems possess an unparalleled ability to safely and environmentally produce complex metabolites and high-value molecules, a capability that is not easily matched by microbial systems. As a result, plant-based production systems, with their unique advantages, may represent a more suitable and effective approach in certain contexts.

In plant synthetic biology, selecting an appropriate host for target compound production is often more crucial than optimizing molecular parts and enzymes. Various plant species, including *N. benthamiana*, *Oryza sativa*, *Solanum lycopersicum*, *Zea mays, Physcomitrella patens*, and *Arabidopsis thaliana*, have been utilized in metabolic engineering and synthetic biology systems (Zhu et al., 2021; Liu et al., 2023; Lin et al., 2023). In recent years, *N. benthamiana* has emerged as a common model plant in synthetic biology due to its high biomass yield, short growth cycle, and efficient genetic transformation techniques, both transient and stable (Lin et al., 2023; Liu et al., 2025). Additionally, the choice of plant species and specific organs—such as leaves, fruits, seeds, hairy roots, or suspension cells—depends on the target compound to be produced. Suitable host selection criteria include a short life cycle, minimal environmental influence, and reduced biosafety concerns. Furthermore, selecting plant hosts with abundant precursor substrates and rich metabolic diversity can be advantageous for enhancing biosynthetic efficiency. In conclusion, further studies should be conducted to select and develop suitable plant hosts to enhance the production of target compounds. Genetically engineered plants allow for the tailored biosynthesis of specific compounds, reducing the formation of by-products and waste and facilitating downstream purification processes. Furthermore, cultivating these plants in indoor farm or vertical farm facility would provide independence from seasonal, climate, or geographical variations. Thus, these integrated systems could be referred to as plant biofactory. Plant biofactory offers several advantages compared to chemical synthesis (Fan, 2024). They operate at low temperatures and atmospheric pressures and do not require very expensive facilities, do not require chemical catalysts, do not pollute the environment, and can reduce production costs. Therefore, plant biofactory could represent a sustainable system for biomolecule production in the future (Huebbers and Buyel, 2021; Wu et al., 2021; Sonkar et al., 2023).

Flavonoid biosynthetic pathways in plants are well established, and many molecular parts are biochemically well characterized. Several flavonoids have already been successfully produced through metabolic engineering in plants using synthetic biology (Lee et al., 2024). Given their diverse applications, flavonoids represent practical, model targets for production in plant biofactory using synthetic biology.

## 5 Conclusions

Flavonoids are valuable secondary metabolites derived from several different kinds of plants. To produce flavonoids in heterologous plant system using plant synthetic biology, first of all we have to get the biochemical information regarding the enzymes in biosynthetic pathway. We collected and standardized experimental enzyme kinetics data for more than 90 cases of nine enzymes such as PAL, C4H, 4CL, CHS, CHI, FNS, F3H, FLS and DFR involved in flavonoid biosynthesis from various plant species. And then we compared their enzyme efficiencies (Kcat/Km) or Michaelis constant (Km) and selected 2 ~ 3 enzymes having the most efficient activity or the highest substrate affinity among each plant-derived enzymes. Those enzymes could serve as the best components for production of flavonoid using plant synthetic biology. These selected components might be valuable parts for plant biofactories to produce flavonoids using plants as hosts.
