# Supplementary material for: Biochemical evaluation of molecular parts for flavonoid production using plant synthetic biology
Source: Front Plant Sci. 2025 Apr 15;16:1528122. doi: 10.3389/fpls.2025.1528122 (PMC12038446; doi:10.3389/fpls.2025.1528122)
Supplement: Supplementary file 1 [file Table1.docx]

***Supplementary Material***

**Supplementary Table 1. Parts characterized biochemically for phenylpropanoid biosynthetic pathway**

| Enzymes | Species | Gene  name | ID | Substrates | K_m_  (μM) | V_max_ (nKat∙mg^-1^) | Kcat | K_cat_/K_m_ (M^-1^∙S^-1^) | V_max_/K_m_ | References |
| --- | --- | --- | --- | --- | --- | --- | --- | --- | --- | --- |
| PAL | *Arabidopsis thaliana* | AtPAL1 | At2g37040 | L-Phe | 68 | 5.5 | 1.8 | 26,471 |  | Cochrane et al., 2004 |
|  |  | AtPAL2 | At3g53260 | L-Phe | 64 | 10.5 | 3.2 | 50,000 |  |  |
|  |  | AtPAL3 | At5g04230 | L-Phe | 2,560 | 0.4 | 0.1 | 39 |  |  |
|  |  | AtPAL4 | At3g10340 | L-Phe | 71 | 9.9 | 3 | 42,254 |  |  |
|  | *Nicotiana tabacum* | PAL1 | M84466 | L-Phe | 59.8 | 14 | 1.09 | 18,227 |  | Reichert et al., 2009 |
|  |  | PAL2 | D17467 | L-Phe | 39.5 | 14.7 | 1.14 | 28,861 |  |  |
|  |  | PAL3 | X78269 | L-Phe | 36.4 | 9.8 | 0.78 | 21,429 |  |  |
|  |  | PAL4 | EU883669/70 | L-Phe | 52.4 | 19.6 | 1.53 | 29,198 |  |  |
|  | *Pinus yunnanensis* | PyPAL1 | OR714894 | L-Phe | 1.861  (mmol∙L^-1^) | 28.8 |  |  | 15.48 | Mu et al., 2024 |
|  |  | PyPAL2 | OR714895 | L-Phe | 2.364  (mmol∙L^-1^) | 22.2 |  |  | 9.39 |  |
|  |  | PyPAL3 | OR714896 | L-Phe | 1.049  (mmol∙L^-1^) | 8.827 |  |  | 8.41 |  |
|  | *Sorghum bicolor* | SbPAL1 | Sb04g026510 | L-Phe | 340 |  | 1.76 | 5,176 |  | Jun et al. 2018 |
|  |  |  |  | L-Tyr | 120 |  | 0.31 | 2,583 |  |  |
|  |  |  |  | L-DOPA | 400 |  | 0.3 | 750 |  |  |
|  |  |  | Sb06g022740 | L-Phe | 4,020 |  | 2.34 | 582 |  |  |
|  |  |  |  | L-Tyr | 420 |  | 0.37 | 881 |  |  |
|  |  |  | Sb04g026520 | L-Phe | 1,520 |  | 2.2 | 1,447 |  |  |
|  |  |  |  | L-Tyr | NA |  | NA | NA |  |  |
|  | *Zea may* | ZmPAL1 | L77912 | L-Phe | 658 |  | 11.9 | 18,085 |  | Rösler et al. 1997 |
|  |  |  |  | L-Tyr | 41 |  | 0.94 | 22,927 |  |  |
| C4H | *Glycine max* | GmC4H2 | Glyma.02G236500 | trans-Cinnamic acid | 6.438 | 0.060 |  |  | 0.009 | Khatri et al. 2023 |
|  |  | GmC4H14 | Glyma.14G205200 | trans-Cinnamic acid | 2.74 | 0.940 |  |  | 0.343 |  |
|  |  | GmC4H20 | Glyma.20G114200 | trans-Cinnamic acid | 3.83 | 0.002 |  |  | 0.001 |  |
|  | *Sorghum bicolor* | SbC4H1 | Sobic.002G126600 | trans-Cinnamic acid | 0.61 | 12.1  (min^-1^) |  |  | 19.836 | Zhang et al., 2020 |
|  |  |  |  | 2-Naphthoic acid | 0.76 | 7.7  (min^-1^) |  |  | 10.132 |  |
|  | *Pyrus bretschneideri* | PbC4H1 | Pbr013141.1  Pbr017290.1 | trans-Cinnamic acid  trans-Cinnamic acid | 10.23  12.46 |  |  |  |  | Li et al., 2020a |
|  |  | PbC4H3 |  |  |  |  |  |  |  |  |
|  | *Leucojum aestivum* | LaeC4H | UIP35210 | trans-Cinnamic acid | 1.21 | 0.12  (μM∙min^-1^) |  |  | 0.099 | Karimzadegan et al., 2024 |
|  | *Populus trichocarpa* | PtrC4H1 | POPTR  _0013s15380  POPTR  _0019s15110 | trans-Cinnamic acid  trans-Cinnamic acid  trans-Cinnamic acid | 7.05  40.68  3.69 | 39.83  267.67  1652.83 |  |  | 5.65  6.58  447.92 | Chen et al., 2011 |
|  |  | PtrC4H2 |  |  |  |  |  |  |  |  |
|  |  | PtrC4H1  +PtrC4H2 |  |  |  |  |  |  |  |  |
| 4CL | *Arabidopsis thaliana* | At4CL1 | At1g51680 | Cinnamic acid | 135 | 9.2 | 0.59 | 4,370 |  | Costa et al., 2005 |
|  |  |  |  | p-Coumaric acid | 6 | 61.3 | 3.96 | 660,000 |  |  |
|  |  |  |  | Caffeic acid | 2 | 42 | 2.71 | 1,355,000 |  |  |
|  |  |  |  | Ferulic acid | 16 | 39.5 | 2.55 | 159,375 |  |  |
|  |  |  |  | 5-OH ferulic acid | 21 | 10.2 | 0.66 | 31,429 |  |  |
|  |  |  |  | Sinapic acid | NC |  |  |  |  |  |
|  |  | At4CL2 | At3g21240 | Cinnamic acid | 181 | 3.2 | 0.21 | 1,160 |  |  |
|  |  |  |  | p-Coumaric acid | 45 | 46.6 | 3 | 66,667 |  |  |
|  |  |  |  | Caffeic acid | 4 | 30.4 | 1.96 | 490,000 |  |  |
|  |  |  |  | Ferulic acid | 686 | 9.9 | 0.64 | 933 |  |  |
|  |  |  |  | 5-OH ferulic acid | 142 | 7 | 0.45 | 3,169 |  |  |
|  |  |  |  | Sinapic acid | NC |  |  |  |  |  |
|  |  | At4CL3 | At1g65060 | Cinnamic acid | 42 | 4 | 0.26 | 6,190 |  |  |
|  |  |  |  | p-Coumaric acid | 4 | 12.9 | 0.84 | 210,000 |  |  |
|  |  |  |  | Caffeic acid | 13 | 7 | 0.45 | 34,615 |  |  |
|  |  |  |  | Ferulic acid | 10 | 6.4 | 0.41 | 41,000 |  |  |
|  |  |  |  | 5-OH ferulic acid | 37 | 0.9 | 0.06 | 1,622 |  |  |
|  |  |  |  | Sinapic acid | NC |  |  |  |  |  |
| 4CL | *Arabidopsis thaliana* | At4CL4 | At3g21230 | Cinnamic acid | NC |  |  |  |  | Costa et al., 2005 |
|  |  |  |  | p-Coumaric acid | 25 | 3.1 | 0.2 | 8,000 |  |  |
|  |  |  |  | Caffeic acid | 7 | 2.9 | 0.19 | 27,143 |  |  |
|  |  |  |  | Ferulic acid | 6 | 0.9 | 0.06 | 10,000 |  |  |
|  |  |  |  | 5-OH ferulic acid | 6 | 2.5 | 0.17 | 28,333 |  |  |
|  |  |  |  | Sinapic acid | 10 | 1.1 | 0.08 | 8,000 |  |  |
|  | *Oryza sativa* L. ssp japonica | Os4CL1 | Os08g14760 | Cinnamic acid | 9.4 | 0.1 | 0.006 | 656 |  | Gui et al., 2011 |
|  |  |  |  | p-Coumaric acid | 11.9 | 0.13 | 0.008 | 686 |  |  |
|  |  |  |  | Caffeic acid | 29.3 | 0.14 | 0.009 | 296 |  |  |
|  |  |  |  | Ferulic acid | 8.3 | 0.11 | 0.007 | 823 |  |  |
|  |  |  |  | Sinapic acid | NC |  |  |  |  |  |
|  |  | Os4CL2 | Os02g46970 | Cinnamic acid | 21.7 | 0.299 | 0.019 | 868 |  |  |
|  |  |  |  | p-Coumaric acid | 16.8 | 0.629 | 0.040 | 2,351 |  |  |
|  |  |  |  | Caffeic acid | 27.6 | 0.708 | 0.045 | 1,612 |  |  |
|  |  |  |  | Ferulic acid | 2.2 | 0.613 | 0.039 | 17,500 |  |  |
|  |  |  |  | Sinapic acid | NC |  |  |  |  |  |
|  |  | Os4CL3 | Os02g08100 | Cinnamic acid | 28.2 | 3 | 0.182 | 6,454 |  |  |
|  |  |  |  | p-Coumaric acid | 4.9 | 5 | 0.285 | 58,163 |  |  |
|  |  |  |  | Caffeic acid | 10.9 | 4 | 0.255 | 23,394 |  |  |
|  |  |  |  | Ferulic acid | 3.5 | 4.93 | 0.299 | 85,429 |  |  |
|  |  |  |  | Sinapic acid | NC |  |  |  |  |  |
|  |  | Os4CL4 | Os06g44620 | Cinnamic acid | 15.7 | 0.35 | 0.021 | 1,359 |  |  |
|  |  |  |  | p-Coumaric acid | 3.9 | 0.77 | 0.047 | 12,051 |  |  |
|  |  |  |  | Caffeic acid | 5.8 | 0.59 | 0.036 | 6,207 |  |  |
|  |  |  |  | Ferulic acid | 4.6 | 0.52 | 0.032 | 6,957 |  |  |
|  |  |  |  | Sinapic acid | NC |  |  |  |  |  |
|  |  | Os4CL5 | Os08g34790 | Cinnamic acid | 54.4 | 0.3 | 0.017 | 313 |  |  |
|  |  |  |  | p-Coumaric acid | 10.3 | 0.83 | 0.048 | 4,660 |  |  |
|  |  |  |  | Caffeic acid | 26.1 | 0.24 | 0.014 | 536 |  |  |
|  |  |  |  | Ferulic acid | 6.9 | 0.59 | 0.034 | 4,928 |  |  |
|  |  |  |  | Sinapic acid | 58.9 | 0.92 | 0.053 | 900 |  |  |
| 4CL | *Glycine max* | Gm4CL1 | AF279267 | Cinnamic acid | 4,400 | 9 % * |  |  | 2X10^-3^ | Lyndermayr et al., 2002 |
|  |  |  |  | p-Coumaric acid | 22 | 100 % * |  |  | 4.54 |  |
|  |  |  |  | Caffeic acid | 33 | 40 % * |  |  | 1.21 |  |
|  |  |  |  | Ferulic acid | 8 | 57 % * |  |  | 7.13 |  |
|  |  |  |  | Sinapic acid | 11 | 35 % * |  |  | 3.21 |  |
|  |  |  |  | 3,pDimethoxycinnamic acid | 83 | 75% * |  |  | 0.91 |  |
|  |  | Gm4CL2 | AF002259 | Cinnamic acid | 1,700 | 50 % * |  |  | 0.03 |  |
|  |  |  |  | p-Coumaric acid | 42 | 100 % * |  |  | 2.38 |  |
|  |  |  |  | Caffeic acid | 13 | 37 % * |  |  | 2.85 |  |
|  |  |  |  | Ferulic acid | 140 | 71 % * |  |  | 0.51 |  |
|  |  |  |  | Sinapic acid | NC |  |  |  |  |  |
|  |  |  |  | 3,pDimethoxycinnamic acid | NC |  |  |  |  |  |
|  |  | Gm4CL3 | AF002258 | Cinnamic acid | 1,100 | 45 % * |  |  | 0.04 |  |
|  |  |  |  | p-Coumaric acid | 9 | 100 % * |  |  | 11.12 |  |
|  |  |  |  | Caffeic acid | 50 | 74 % * |  |  | 1.48 |  |
|  |  |  |  | Ferulic acid | 3,100 | 25 % * |  |  | 8.1X10^-3^ |  |
|  |  |  |  | Sinapic acid | NC |  |  |  |  |  |
|  |  |  |  | 3,pDimethoxycinnamic acid | NC |  |  |  |  |  |
|  |  | Gm4CL4 | X69955 | Cinnamic acid | 260 | 20 % * |  |  | 0.08 |  |
|  |  |  |  | p-Coumaric acid | 10 | 100 % * |  |  | 10 |  |
|  |  |  |  | Caffeic acid | 34 | 50 % * |  |  | 1.47 |  |
|  |  |  |  | Ferulic acid | 1,300 | 30 % * |  |  | 0.02 |  |
|  |  |  |  | Sinapic acid | NC |  |  |  |  |  |
|  |  |  |  | 3,pDimethoxycinnamic acid | NC |  |  |  |  |  |

No conversion (NC).

Relative percentage (%) of p-Coumaric acid (*).

**Supplementary Table 2. Parts characterized biochemically for flavonoid biosynthetic pathway**

| Enzymes | Species | Gene  name | ID | Substrates | K_m_  (μM) | V_max_ (nKat∙mg^-1^) | K_cat_  (S^-1^) | K_cat_/K_m_  (M^-1^∙S^-1^) | V_max_/K_m_ | References |
| --- | --- | --- | --- | --- | --- | --- | --- | --- | --- | --- |
| CHS | *Oryza sativa* L.  *ssp japonica* | OsCHS8 | Os07g0214900 | p-coumaroyl  -CoA | 27.6 | 0.00587 | 0.00025 | 9 |  | Park et al., 2020 |
|  |  | OsCHS24 | Os11g0529500 | p-coumaryol  -CoA | 45.4 | 0.02030 | 0.00086 | 19 |  |  |
|  | *Physcomitrella patens* | PpCHS | Pp3c24_13120 | p-coumaroyl  -CoA | 19.0 |  | 0.02817 | 1,482 |  | Waki et al., 2020 |
|  | *Selaginella moellendorffii* | SmCHS | 270496 | p-coumaroyl  -CoA | 3.15 |  | 0.06567 | 20,847 |  |  |
|  | *Ginko*  *biloba* | GbCHS | AY647263 | p-coumaroyl  -CoA | 4.29 |  | 0.11317 | 26,379 |  |  |
|  | *Oryza*  *sativa* | OsCHS-1 | LOC_Os11g32650 | p-coumaroyl  -CoA | 7.7 |  | 0.02633 | 3,420 |  |  |
|  | *Antirrhinum*  *majus* | AmCHS | X03710 | p-coumaroyl  -CoA | 4.73 |  | 0.08717 | 18,428 |  |  |
|  | *Medicago sativa Alfalfa* | CHS2 | P30074 | p-Coumaroyl  -CoA | 6.1 |  | 0.08567 | 14,044 |  | Jez et al., 2001 |
|  | *Humulus*  *lupulus* | CHS H1 | AJ304877 | p-coumaroyl  -CoA | 40.9 |  |  |  |  | Novak et al., 2006 |
|  |  | VPS (valerophenone synthase) | AB047593 | p-coumaroyl  -CoA | 29.0 |  |  |  |  |  |
|  | *Pueraria*  *lobata* | CHS | P23569 | p-Coumaroyl  -CoA | 50.0 |  |  |  |  | Fukuma et al., 2007 |
| CHI | *Glycine*  *max* | GmCHI2 | Glyma.20G241700 | Naringenin chalcone | 2.00 |  | 478.8 | 239,400,000 |  | Ralston et al., 2005 |
|  |  | GmCHI1A | Glyma.20G241500 | Naringenin chalcone | 15.0 |  | 152.2 | 10,146,667 |  |  |
|  |  | GmCHI1B2 | Glyma.10G29220 | Naringenin chalcone | 7.0 |  | 219.1 | 31,300,000 |  |  |
|  | *Oryza sativa*  *spp japonica* | OsCHI3 | Os03g0819600 | Naringenin chalcone | 11.6 | 2,662 | 69.3 | 5,978,448 |  | Park et al., 2021 |
| CHI | *Medicago sativa*  *Alfalfa* | MsCHI1 | P28012 | Narigenin  chalcone | 53.6 | 4329 | 103.0 | 1,920,348 |  | Cheng et al., 2018 |
|  | *Marchantia paleacea* | MpCHI1 | KT428982 | Naringenin chalcone | 53.5 | 3898 | 108.7 | 2,031,562 |  |  |
|  | *Plagiochasma appendiculatum* | PaCHI1 | KT428984 | Naringenin chalcone | 77.5 | 3330 | 75.6 | 975,232 |  |  |
|  | *Selaginella moellendorffii* | SmCHI1 | XP_002987458 | Naringenin chalcone | 84.7 | 3048 | 70.4 | 830,424 |  |  |
|  | *Deschampsia antarcita* | DaCHI1 | FR714890.1 | Naringenin chalcone | 8.50 |  | 130.3 | 15,331,373 |  | Park et al., 2018 |
| F3H | *Arabidopsis thaliana* | AtF3H/TT6 | At3g51240 | Naringenin | 24.0 | 0.000002 |  |  | 0.00000007 | Owens et al., 2008 |
|  | *Carthamus tinctorius* L. (safflower) | CtF3H | AEG64806.1 | Naringenin | 43.7 | 0.000382 |  |  |  | Tu et al., 2016 |
|  | *Glycine*  *max* | GmF3H1 | AY669325, Glyma.02g0484800V4 | Eriodictyol | 87.0 | 0.1024 |  |  | 0.00117 | Kim et al., 2008a |
|  | *Oryza*  *sativa* | OsF3H1 | NM_001060692 Os02g0767300 | Eriodictyol | 57.8 | 0.0031 | 0.21 | 3,633 | 0.00005 | Kim et al., 2008b |
|  |  | OsF3H-2 | AAL58118 Os10g0536400 | Eriodictyol | 5.7 | 0.4 | 0.00024 | 42 | 0.070 |  |
|  |  | OsF3H-3 | CAE02796 Os04g0667200 | Eriodictyol | 6.3 | 0.5 | 0.003 | 476 | 0.079 |  |
| FNSI | *Plagiochasma appendiculatum* | PaFNSI  /H2H | KJ439220 | Naringenin | 19.9 |  | 0.027 | 1,356 |  | Han et al., 2014 |
|  |  | PaFNSI  /H2HY240P | KJ439220 | Naringenin | 6.5 |  | 0.002 | 307 |  |  |
|  | *Conocephalum japonicum* | CjFNSI1 | MK557768 | Naringenin | 9.4 |  | 0.250 | 26,596 |  | Li et al., 2020b |
|  |  |  |  | DHK | 13.0 |  | 0.300 | 23,076 |  |  |
|  |  | CjFNSI1  /F2H | MK557767 | Naringenin | 14.0 |  | 0.170 | 12,143 |  |  |
|  |  |  |  | DHK | 38.0 |  | 0.042 | 1,105 |  |  |
|  | *Physcomitrella patens* | PpFNSI  /F3H | XP_001780809 | Naringenin | 95.0 |  | 0.260 | 2,737 |  |  |
|  | *Selaginella moellendorffii* | SmFNSI  /F3H | XP_002985262 | Naringenin | 91.0 |  | 0.330 | 3,626 |  |  |
|  | *Petroselinum crispum* | PcFNSI | AY817680 | Naringenin | 0.31 |  | 0.027 | 87,097 |  |  |
| FNSI | *Zea*  *mays* | ZmFNSI-1 | GRMZM2G09967 | Naringenin |  | 0.257 |  |  |  | Ferreyra et al., 2015 |
|  | *Arabidopsis thaliana* | AtFNSI/AtDMR6 | At05g08640 | Naringenin |  | 0.181 |  |  |  |  |
|  | *Oryza*  *sativa* | OsFNSI-1 | 37536443 | Naringenin | 29.0 | 43 |  |  | 1.48 | Lee et al., 2008 |
| FNSII | *Lonicera*  *japonica* | LjFNSII  /F2H-1.1 | KU127576 | Naringenin | 9.93 | 13.8 |  |  | 1.39 | Wu et al., 2016 |
|  |  |  |  | Liquiritigenin | 6.48 | 38.4 |  |  | 5.92 |  |
|  |  | LjFNSII  /F2H -2.1 | KU127578 | Naringenin | 1.63 | 2.42 |  |  | 1.48 |  |
|  | *Lonicera macranthoides* | LmFNSII  /F2H -1.1 | KU127580 | Naringenin | 1.63 | 1.73 |  |  | 1.06 |  |
|  | *Perilla*  *frutescens* | PfFNSII | BAB59004.1 | Naringenin | 8.8 | 0.0048 |  |  | 0.00054545 | Kitada et al., 2001 |
|  | *Gentiana*  *triflora* | GtFNSII | AB193314 | Naringenin | 8.9 | 0.0049 |  |  | 0.00055056 | Nakatsuka et al., 2006 |
|  | *Oryza*  *sativa* | OsFNSII-1 | Os04g01140 | Naringenin | 3.20 | 0.000019 |  |  | 0.00000594 | Lam et al., 2014 |
|  | *Glycine*  *max* | GmFNSII | Glyma  12g07190.01 | Naringenin | 2.50 |  |  |  |  | Fliegmann et al., 2010 |
|  |  |  |  | Eriodictyol | 1.80 |  |  |  |  |  |
|  |  |  |  | Liquiritigenin | 4.20 |  |  |  |  |  |
|  | *Fortunella crassifolia* | FcFNSII-2 | Sjg260830.1 | Naringenin | 3.77 | 0.000012 |  |  | 0.00000318 | Tian et al., 2022 |
| FLS | *Populus*  *deltoides* | PdFLS | TC74233 | DHK | 24.0 |  |  |  |  | Kim et al., 2010 |
|  |  |  |  | DHQ | 31.0 |  |  |  |  |  |
|  | *Alium*  *cepa* | AcFLS-H6 | KY369209 | DHK | 20.3 |  | 0.0014 | 69 |  | Park et al., 2017 |
|  |  |  |  | DHQ | 24.4 |  | 0.0096 | 393 |  |  |
|  |  | AcFLS-HRB | KY369210 | DHK | 15.5 |  | 0.0024 | 155 |  |  |
|  |  |  |  | DHQ | 26.3 |  | 0.0211 | 802 |  |  |
|  | *Arabidopsis thaliana* | AtFLS1 | At05g08640 | DHQ | 59.0 |  | 0.0560 | 949 |  | Chua et al., 2008 |
|  |  | AtFLS1H132F | At05g08640 | DHQ | 27.0 |  | 0.0340 | 1259 |  |  |
| FLS | *Ornithogalum caudatum* | OcFLS1 | MH748569 | DHK | 471.3 | 214 (nkat) |  |  | 0.454 | Sun et al., 2019 |
|  |  |  |  | Naringenin | 37.6 | 34 (nkat) |  |  | 0.910 |  |
|  |  | OcFLS2 | MH748570 | DHK | 525.5 | 251 (nkat) |  |  | 0.478 |  |
|  |  |  |  | Naringenin | 110.5 | 96 (nkat) |  |  | 0.877 |  |
|  | *Ginkgo*  *biloba* | GbFLS | GQ994432 | DHK | 74.0 | 305 (nkat) |  |  | 4.12 | Xu et al., 2012 |
|  | *Camellia*  *sinensis* | CsFLS | EF205150 | DHQ | 139.0 |  | 0.16 | 1,151 |  | Lin et al., 2007 |
|  | *Zea*  *mays* | ZmFLS1 | BT039956 | DHK | 58.4 |  | 6.6 | 113,014 |  | Ferreyra et al., 2010 |
|  |  |  |  | DHQ | 151.1 |  | 3.9 | 25,811 |  |  |
|  | *Rubus*  *chingii* | RcFLS1 | LG02.1317 | DHK | 33.9 |  | 0.0749 | 2,209 |  | Lei et al., 2023 |
|  |  |  |  | DHQ | 56.9 |  | 0.1489 | 2,617 |  |  |
|  |  |  |  | Naringenin | 43.6 |  | 0.0045 | 103 |  |  |
|  |  |  |  | Eriodictyol | 34.5 |  | 0.0143 | 414 |  |  |
| DFR | *Rubus*  *chingii* | RcDFRS | LG07.3952 | DHK | 47.2 |  | 0.0805 | 1,706 |  | Lei et al., 2023 |
|  |  |  |  | DHQ | 124.9 |  | 0.1056 | 845 |  |  |
|  | *Camelina*  *sinensis* | CsDFRa | KY615690 | DHK | 145.1 |  | 5.41 | 37,285 |  | Ruan et al., 2022 |
|  |  |  |  | DHQ | 41.8 |  | 10.39 | 248,565 |  |  |
|  |  |  |  | DHM | 58.4 |  | 10.47 | 179,158 |  |  |
|  |  | CsDFRc | KY615694 | DHK | 42.3 |  | 5.74 | 135,665 |  |  |
|  |  |  |  | DHQ | 81.8 |  | 7.21 | 88,142 |  |  |
|  |  |  |  | DHM | 105.6 |  | 15.24 | 144,373 |  |  |
|  |  | CsDFR  b1N120S | KY615694 | DHK | 149.0 |  | 6.76 | 45,381 |  |  |
|  |  |  |  | DHQ | 108.7 |  | 10.97 | 100,957 |  |  |
|  |  |  |  | DHM | 96.6 |  | 8.51 | 88,113 |  |  |
|  |  | CsDFR  b1C126T | KY615694 | DHK | 131.1 |  | 6.66 | 50,805 |  |  |
|  |  |  |  | DHQ | 102.6 |  | 6.66 | 64,931 |  |  |
|  |  |  |  | DHM | 47.8 |  | 8.95 | 187,435 |  |  |
|  | *Fragaria Xananassa*  *cv. Elsanta* | DFR1 ES | KC894048 | DHK | 0.40 | 11.4 |  |  | 28.5 | Miosic et al., 2014 |
|  |  | DFR2 ES | KC894055 | DHQ | 0.40 | 3.1 |  |  | 7.75 |  |
|  |  |  |  | DHM | 2.30 | 11.2 |  |  | 4.87 |  |

**References**

Chen, H.C., Li, Q., Shuford, C.M., Liu, J., Muddiman, D.C., Sederoff, R.R., et al. (2011). Membrane protein complexes catalyze both 4- and 3-hydroxylation of cinnamic acid derivatives in monolignol biosynthesis. *Proc. Natl. Acad. Sci. U.S.A.* 108, 21253-21258. doi: 10.1073/pnas.1116416109

Costa, M.A., Bedgar, D.L., Moinuddin, S.G., Kim, K.W., Cardenas, C.L., Cochrane, F.C., et al. (2005). Characterization in vitro and in vivo of the putative multigene 4-coumarate: CoA ligase network in Arabidopsis: syringyl lignin and sinapate/sinapyl alcohol derivative formation. Phytochemistry 66, 2072-2091. doi: 10.1016/j.phytochem.2005.06.022

Cheng, A.X., Zhang, X., Han, X.J., Zhang, Y.Y., Gao, S., Liu, C.J., et al. (2018). Identification of chalcone isomerase in the basal land plants reveals an ancient evolution of enzymatic cyclization activity for synthesis of flavonoids. *New Phytol.* 217, 909-924. doi: 10.1111/nph.14852

Chua, C.S., Biermann, D., Goo, K.S., and Sim, T.S. (2008). Elucidation of active site residues of Arabidopsis thaliana flavonol synthase provides a molecular platform for engineering flavonols. *Phytochemistry* 69, 66-75. doi: 10.1016/j.phytochem.2007.07.006
Cochrane, F.C., Davin, L.B., and Lewis, N.G. (2004). The Arabidopsis phenylalanine ammonia lyase gene family: kinetic characterization of the four PAL isoforms. *Phytochemistry* 65, 1557-1564. doi: 10.1016/j.phytochem.2004.05.006

Cochrane, F.C., Davin, L.B., and Lewis, N.G. (2004). The Arabidopsis phenylalanine ammonia lyase gene family: kinetic characterization of the four PAL isoforms. *Phytochemistry* 65, 1557-1564. doi: 10.1016/j.phytochem.2004.05.006

Costa, M.A., Bedgar, D.L., Moinuddin, S.G., Kim, K.W., Cardenas, C.L., Cochrane, F.C., et al. (2005). Characterization in vitro and in vivo of the putative multigene 4-coumarate: CoA ligase network in Arabidopsis: syringyl lignin and sinapate/sinapyl alcohol derivative formation. *Phytochemistry* 66, 2072-2091. doi: 10.1016/j.phytochem.2005.06.022

Cui, Y., and Li, J. (2019). Characterization of a dihydroflavonol 4-reductase gene in safflower and its potential role in flavonoid biosynthesis. *Plant Cell Rep.* 38, 125-135. doi: 10.1007/s00299-018-2385-8

Chua, C.S., Biermann, D., Goo, K.S., and Sim, T.S. (2008). Elucidation of active site residues of Arabidopsis thaliana flavonol synthase provides a molecular platform for engineering flavonols. Phytochemistry 69, 66-75. doi: 10.1016/j.phytochem.2007.07.006

Ferreyra, M.L.F, Emiliani, J., Rodriguez, E.J., Campos-Bermudez, V.A., Grotewold, E., and Casati, P. (2015). The identification of maize and Arabidopsis type I FLAVONE SYNTHASEs links flavones with hormones and biotic interactions. *Plant Physiol.* 169, 1090-1107. doi: 10.1104/pp.15.00515

Fliegmann, J., Furtwängler, K., Malterer, G., Cantarello, C., Schüler, G., Ebel, J., et al. (2010). Flavone synthase II (CYP93B16) from soybean (Glycine max L.). *Phytochemistry* 71, 508-514. doi: 10.1016/j.phytochem.2010.01.007

Fukuma, K., Neuls, E. D., Ryberg, J. M., Suh, D. Y., and Sankawa, U. (2007). Mutational analysis of conserved outer sphere arginine residues of chalcone synthase. *J. Biochem.* 142, 731–739. doi: 10.1093/jb/mvm188

Gosch, C., Halbwirth, H., Stich, K., and Forkmann, G. (2014). Dihydroflavonol 4-reductase from *Malus domestica* and its role in flavonoid biosynthesis. *J. Plant Physiol.* 171, 1557-1565. doi: 10.1016/j.jplph.2014.06.013

Gui, J., Shen, J., and Li, L. (2011). Functional characterization of evolutionarily divergent 4-coumarate: Coenzyme A ligases in rice. *Plant Physiol.* 157, 574-586. doi: 10.1104/pp.111.178301

Han, X. J., Wu, Y. F., Gao, S., Yu, H. N., Xu, R. X., Lou, H. X., et al. (2014). Functional characterization of a *Plagiochasma appendiculatum* flavone synthase I showing flavanone 2-hydroxylase activity. *FEBS Lett.* 588, 2307-2314. doi: 10.1016/j.febslet.2014.05.023

Jeong, Y., Lee, M. H., Kim, H., Hwang, J., and Park, S. U. (2014). Characterization and expression analysis of dihydroflavonol 4-reductase gene in *Chrysanthemum morifolium*. *Genes Genomics* 36, 347-354. doi: 10.1007/s13258-014-0154-y

Jez, J.M., Bowman, M.E., and Noel, J.P. (2001). Structure and mechanism of the evolutionarily unique plant enzyme chalcone isomerase. *Biochemistry* 40, 14829-14838.

Jun, S.Y., Sattler, S.A., Cortez, G.S., Vermerris, W., Sattler, S.E., and Kang, C.H. (2018). Biochemical and structural analysis of substrate specificity of a phenylalanine ammonia-lyase. *Plant Physiol.* 176, 1452-1468. doi: 10.1104/pp.17.01608

Karimzadegan, V., Koirala, M., Sobhanverdi, S., Merindol, N., Majhi, B. B., Gélinas, S. E., Timokhin, V. I., Ralph, J., Dastmalchi, M., and Desgagné-Penix, I. (2024). Characterization of cinnamate 4-hydroxylase (CYP73A) and p-coumaroyl 3′-hydroxylase (CYP98A) from *Leucojum aestivum*, a source of Amaryllidaceae alkaloids. *Plant Physiol. Biochem.* 210. doi: 10.1016/j.plaphy.2024.108612

Kitada, C., Gong, Z., Tanaka, Y., Yamazaki, M., and Saito, K. (2001). Differential expression of two cytochrome P450 involved in the biosynthesis of flavones and anthocyanins in chemo-varietal forms of Perilla frutescens. *Plant Cell Physiol.* 42, 1338-1344. doi: 10.1093/pcp/pce169

Khatri, P., Chen, L., Rajcan, I., and Dhaubhadel, S. (2023). Functional characterization of Cinnamate 4-hydroxylase gene family in soybean (*Glycine max*). *PLoS One* 18, 1–19. doi: 10.1371/journal.pone.0285698

Kim, B. G., Joe, E. J., and Ahn, J. H. (2010). Molecular characterization of flavonol synthase from poplar and its application to the synthesis of 3-O-methylkaempferol. *Biotechnol. Lett.* 32, 579-584. doi: 10.1007/s10529-009-0188-x

Kim, B. G., Kim, J. H., Kim, J., Lee, C., and Ahn, J. H. (2008a). Accumulation of flavonols in response to ultraviolet-B irradiation in soybean is related to induction of flavanone 3-β-hydroxylase and flavonol synthase. *Mol. Cells* 25, 247–252. doi: 10.1016/s1016-8478(23)17577-7

Kim, J.H., Lee, Y.J., Kim, B.G., Lim, Y., and Ahn, J.H. (2008b). Flavanone 3β-hydroxylases from rice: Key enzymes for favonol and anthocyanin biosynthesis. *Mol. Cells* 25, 312-316. doi: 10.1016/s1016-8478(23)17587-x

Kitada, C., Gong, Z., Tanaka, Y., Yamazaki, M., and Saito, K. (2001). Differential expression of two cytochrome P450 involved in the biosynthesis of flavones and anthocyanins in chemo-varietal forms of *Perilla frutescens*. *Plant Cell Physiol.* 42, 1338-1344. doi: 10.1093/pcp/pce169

Khatri, P., Chen, L., Rajcan, I., and Dhaubhadel, S. (2023). Functional characterization of Cinnamate 4-hydroxylase gene family in soybean (*Glycine max*). *PLoS One* 18, 1–19. doi: 10.1371/journal.pone.0285698

Lam, P. Y., Zhu, F. Y., Chan, W. L., Liu, H., and Lo, C. (2014). Cytochrome P450 93G1 is a flavone synthase II that channels flavanones to the biosynthesis of tricin O-linked conjugates in rice. *Plant Physiol.* 165, 1315-1327. doi: 10.1104/pp.114.239723

Lee, Y.J., Kim, J.H., Kim, B.G., Lim, Y., and Ahn, J.H. (2008). Characterization of flavone synthase I from rice. *BMB Rep.* 41, 68-71. doi: 10.5483/bmbrep.2008.41.1.68

Lei, T., Huang, J., Ruan, H., Qian, W., Fang, Z., Gu, C., et al. (2023). Competition between FLS and DFR regulates the distribution of flavonols and proanthocyanidins in Rubus chingii Hu. *Front. Plant Sci.* 10:1181011. doi: 10.3389/fpls.2023.1134993

Li, G., Liu, X., Zhang, Y., Muhammad, A., Han, W., Li, D., Cheng, X., and Cai, Y. (2020a). Cloning and functional characterization of two cinnamate 4-hydroxylase genes from *Pyrus bretschneideri*. *Plant Physiol. Biochem.* 156, 135–145. doi: 10.1016/j.plaphy.2020.07.035

Li, D. D., Ni, R., Wang, P. P., Zhang, X. S., Wang, P. Y., Zhu, T. T., et al. (2020b). Molecular basis for chemical evolution of flavones to flavonols and anthocyanins in land plants. *Plant Physiol.* 184, 1731-1743. doi: 10.1104/pp.20.01185

Lin, Y., Qiu, Z., Shi, L., Wu, X., Chen, G., and He, M. (2020). Functional characterization of flavonoid biosynthetic genes in *Cucumis melo* L. *Genes* 11, 359. doi: 10.3390/genes11030359

Liu, J., Ryu, J., Lee, M., and Chen, J. (2015). Functional analysis of key flavonoid biosynthetic genes in *Citrus sinensis* through the study of flavonoid profiles and expression analysis. *J. Plant Physiol.* 176, 93-101. doi: 10.1016/j.jplph.2014.09.015

Lin, G. Z., Lian, Y. J., Ryu, J. H., Sung, M. K., Park, J. S., Park, H. J., et al. (2007). Expression and purification of His-tagged flavonol synthase of Camellia sinensis from Escherichia coli. *Protein Expr. Purif.* 55, 287-292. doi: 10.1016/j.pep.2007.05.013

Lindermayr, C., Möllers, B., Fliegmann, J., Uhlmann, A., Lottspeich, F., Meimberg, H., et al. (2002). Divergent members of a soybean (*Glycine max* L.) 4-coumarate:coenzyme A ligase gene family. *Eur. J. Biochem.* *269*(4), 1304–1315. doi:10.1046/j.1432-1033.2002.02775.x

Luo, Z., and Lu, H. (2021). Functional characterization of flavonoid biosynthetic genes and their regulation in *Vitis vinifera*. *BMC Plant Biol.* 21, 13. doi: 10.1186/s12870-020-02792-9

Ma, X., Song, J., Liu, W., Dong, Z., Zhao, X., and Zhi, Y. (2020). Identification and functional analysis of flavonoid biosynthetic genes in *Cymbidium goeringii*. *Plant Cell Tissue Organ Cult.* 141, 71-80. doi: 10.1007/s11240-020-01824-4

Ma, X., Wang, X., and Li, Q. (2021). Comprehensive profiling and functional characterization of genes involved in flavonoid biosynthesis in *Amaranthus cruentus*. *J. Agric. Food Chem.* 69, 13253-13263. doi: 10.1021/acs.jafc.1c03612

Miyazaki, T., Yamaguchi, S., and Kitamura, K. (2003). Molecular and functional analysis of two dihydroflavonol 4-reductases from *Nicotiana tabacum* involved in anthocyanin biosynthesis. *Plant Cell Physiol.* 44, 1201-1208. doi: 10.1093/pcp/pcg150

Miosic, S., Thill, J., Milosevic, M., Gosch, C., Pober, S., Molitor, C., et al. (2014). Dihydroflavonol 4-reductase genes encode enzymes with contrasting substrate specificity and show divergent gene expression profiles in *Fragaria* species. *PLOS ONE* 9. doi: 10.1371/journal.pone.0112707

Mu, D., Chen, L., Wang, H., Hu, Z., Chen, S., Chen, S., Cai, N., Xu, Y., and Tang, J. (2024). The Identification of Phenylalanine Ammonia-Lyase (PAL) Genes from Pinus yunnanensis and an Analysis of Enzyme Activity in vitro. *Phyton-International J. Exp. Bot.* 93, 503–516. doi: 10.32604/phyton.2024.048786

Nabavi, S.F., Braidy, N., and M. W., and Xu, J. (2020). Dihydroflavonol 4-reductase gene and its functional role in flavonoid biosynthesis in *Camellia sinensis*. *J. Plant Physiol.* 249, 153-166. doi: 10.1016/j.jplph.2020.153166

Nakatsuka, T., Nishihara, M., Mishiba, K., and Yamamura, S. (2006). Heterologous expression of two gentian cytochrome P450 genes can modulate the intensity of flower pigmentation in transgenic tobacco plants. *Mol. Breed.* 17, 91-99. doi: 10.1007/s11032-005-2520-z

Novak, P., Krofta, K., and Matousek, J. (2006). Chalcone synthase homologues from Humulus lupulus: some enzymatic properties and expression. Biol. Plant. 50, 48-54.

Owens, D. K., Crosby, K. C., Runac, J., Howard, B. A., and Winkel, B. S. J. (2008). Biochemical and genetic characterization of Arabidopsis flavanone 3β-hydroxylase. *Plant Physiol. Biochem.* 46, 833–843. doi: 10.1016/j.plaphy.2008.06.004

Park, H. L., Yoo, Y., Bhoo, S. H., Lee, T. H., Lee, S. W., and Cho, M. H. (2020). Two chalcone synthase isozymes participate redundantly in uv-induced sakuranetin synthesis in rice. *Int. J. Mol. Sci.* 21. doi: 10.3390/ijms21113777

Park, S. I., Park, H. L., Bhoo, S. H., Lee, S. W., and Cho, M. H. (2021). Biochemical and Molecular Characterization of the Rice Chalcone Isomerase Family. *Plants (Basel, Switzerland)*, *10*(10), 2064. doi: 10.3390/plants10102064

Park, S. H., Lee, C. W., Cho, S. M., Lee, H., Park, H., Lee, J., and Lee, J. H. (2018). Crystal structure and enzymatic properties of chalcone isomerase from the Antarctic vascular plant Deschampsia antarctica Desv. *PLoS One* 13, 1–17. doi: 10.1371/journal.pone.0192415

Park, S., Kim, D. H., Lee, J. Y., Ha, S. H., and Lim, S. H. (2017). Comparative analysis of two flavonol synthases from different-colored onions provides insight into flavonoid biosynthesis. *J. Agric. Food Chem.* 65, 5287-5298. doi: 10.1021/acs.jafc.7b01036

Ralston, L., Subramanian, S., Matsuno, M., and Yu, O. (2005). Partial reconstruction of flavonoid and isoflavonoid biosynthesis in yeast using soybean type I and type II chalcone isomerases. *Plant Physiol*. 137, 1375-1388. doi: 10.1104/pp.104.054502

Reichert, A. I., He, X. Z., and Dixon, R. A. (2009). Phenylalanine ammonia-lyase (PAL) from tobacco (*Nicotiana tabacum*): characterization of the four tobacco PAL genes and active heterotetrameric enzymes. *The Biochemical journal*, *424*(2), 233–242. doi:10.1042/BJ20090620

Rösler, J., Krekel, F., Amrhein, N., and Schmid, J. (1997). Maize phenylalanine ammonia-lyase has tyrosine ammonia-lyase activity. *Plant Physiol.* 113, 175–179. doi: 10.1104/pp.113.1.175

Ruan, H., Shi, X., Gao, L., Rashid, A., Li, Y., Lei, T., et al. (2022). Functional analysis of the dihydroflavonol 4-reductase family of Camellia sinensis: exploiting key amino acids to reconstruct reduction activity. *Hortic. Res*. 9, 1-13. doi: 10.1093/hr/uhac098

Sun, Y.J., He, J.M., and Kong, J.Q. (2019). Characterization of two flavonol synthases with iron-independent flavanone 3-hydroxylase activity from Ornithogalum caudatum Jacq. *BMC Plant Biol*. 19, 1-15. doi: 10.1186/s12870-019-1787-x

Tanaka, Y., Sasaki, N., and Ohmiya, A. (2008). Biosynthesis of plant pigments: anthocyanins, betalains and carotenoids. *Plant J.* 54, 733-749. doi: 10.1111/j.1365-313X.2008.03447.x
Vogt, T. (2010). Phenylpropanoid biosynthesis. *Mol. Plant* 3, 2-20. doi: 10.1093/mp/ssp106

Tian, S., Yang, Y., Wu, T., Luo, C., Li, X., and Zhao, X. (2022). Functional characterization of a flavone synthase that participates in a kumquat flavone metabolon. *Front. Plant Sci.* 13:826780. doi: 10.1

Tu, Y. H., Liu, F., Guo, D. D., Fan, L. J., Zhu, Z. X., Xue, Y. R., Gao, Y., and Guo, M. L. (2016). Molecular characterization of flavanone 3-hydroxylase gene and flavonoid accumulation in two chemotyped safflower lines in response to methyl jasmonate stimulation. *BMC Plant Biol.* 16, 1–12. doi: 10.1186/s12870-016-0813-5

Waki, T., Mameda, R., Nakano, T., Yamada, S., Terashita, M., Ito, K., et al. (2020). A conserved strategy of chalcone isomerase-like protein to rectify promiscuous chalcone synthase specificity. *Nat. Commun*. 11, 870. doi: 10.1038/s41467-020-14558-9

Wu, T., Kerbler, S.M., Fernie, A.R., and Zhang, Y. (2021). Plant cell cultures as heterologous bio-factories for secondary metabolite production. Plant Commun. 2, 100235. doi: 10.1016/j.xplc.2021.100235

Xu, F., Li, L., Zhang, W., Cheng, H., Sun, N., Cheng, S., et al. (2012). Isolation, characterization, and function analysis of a flavonol synthase gene from Ginkgo biloba. *Mol. Biol. Rep.* 39, 2285-2296. doi: 10.1007/s11033-011-0978-9

Zhang, B., Lewis, K.M., Abril, A., Davydov, D.R., Vermerris, W., Sattler, S.E., et al. (2020). Structure and function of the cytochrome p450 monooxygenase cinnamate 4-hydroxylase from sorghum bicolor. *Plant Physiol*. 183, 957-973. doi: 10.1104/pp.20.00406
